# Theoretical and experimental studies of (In,Ga)As/GaP quantum dots

**DOI:** 10.1186/1556-276X-7-643

**Published:** 2012-11-23

**Authors:** Cedric Robert, Tra Nguyen Thanh, Charles Cornet, Pascal Turban, Mathieu Perrin, Andrea Balocchi, Herve Folliot, Nicolas Bertru, Laurent Pedesseau, Mikhail O Nestoklon, Jacky Even, Jean-Marc Jancu, Sylvain Tricot, Olivier Durand, Xavier Marie, Alain Le Corre

**Affiliations:** 1 Université Européenne de Bretagne, INSA Rennes, France CNRS, UMR 6082 Foton-Ohm, 20 Avenue des Buttes de Coësmes, Rennes, 35708, France; 2 Equipe de Physique des Surfaces et Interfaces, Institut de Physique de Rennes UMR UR1-CNRS 6251, Université de Rennes 1, Rennes Cedex, F-35042, France; 3 Université de Toulouse, INSA-CNRS-UPS, LPCNO, 135 avenue de Rangueil, Toulouse, 31077, France; 4 Ioffe Physico-Technical Institute, Russian Academy of Sciences, St. Petersburg, 194021, Russia

**Keywords:** Quantum dots, Tight-binding, **k·p** simulation, Time-resolved photoluminescence, 78.55.Cr, 78.47.jd, 78.67.Hc

## Abstract

(In,Ga)As/GaP(001) quantum dots (QDs) are grown by molecular beam epitaxy and studied both theoretically and experimentally. The electronic band structure is simulated using a combination of **k·p** and tight-binding models. These calculations predict an indirect to direct crossover with the In content and the size of the QDs. The optical properties are then studied in a low-In-content range through photoluminescence and time-resolved photoluminescence experiments. It suggests the proximity of two optical transitions of indirect and direct types.

## Background

In the context of the monolithic integration of photonics on silicon, the pseudomorphic approach, i.e., growing lattice-matched compounds on Si, is a promising route towards an efficient and long-term stable laser on Si [1]. It should overcome the issue of the dramatic number of crystalline defects due to the large lattice mismatch encountered in the growth of most III-V materials onto Si substrates [2]. Among binary III-V materials, GaP presents the closest lattice constant to Si (0.37% at 300 K). The perfect lattice matching can even be obtained by introducing 2% of nitrogen in GaP. Recently, the epitaxial growth on Si substrate of GaP and GaPN_0.02_ has been greatly improved by several groups [3-5]. Various active zones grown on GaP substrate or on GaPN_0.02_/GaP/Si have been proposed. The best results have been achieved with compressive strained GaNAsP/GaP quantum wells (QWs) in electrically pumped lasers operating up to 150 K (Si substrate) [6] or at room temperature (GaP substrate) [7]. However, the electron wave function at the conduction band minimum has a special character [8]. It is expected to limit the performances of laser devices yielding high threshold current densities. Indeed, the conduction band of the GaAsP host material has a minimum at the *X*_XY_ point on the edge of the Brillouin zone, and partially localized electronic levels related to nitrogen incorporation lie at energies below this minimum. The conduction band minimum of GaNAsP/GaP QWs evidences a predominant localized N character [8]. Moreover, the maximum of the emission wavelength reported for such structures with reasonable N content is equal to 980 nm [7], which is not yet in the transparency window of Si.

Quantum dot (QD) lasers grown on GaAs or InP substrate display lower threshold currents due to the 0D density of states when compared with QW lasers on the same substrates [9]. (In,Ga)P QDs grown on GaP substrate have already been studied, and room temperature electroluminescence has been obtained [10]. However, theoretical studies have shown that the electronic band lineups correspond to a borderline case between type I and type II [11]. The (In,Ga)As(N)/GaP QDs system has recently attracted much attention. Fukami et al. [12] have claimed that the transparency window of silicon may be reached with InGaAsN/GaP QDs when In composition is 50% to approximately 60% and N composition is 1% to approximately 2%. In the following, InGaAs/GaP QDs are studied as a step toward InGaAsN/GaP QDs system. Both room-temperature photoluminescence (PL) [13] and electroluminescence [14] of InGaAs/GaP QDs have been recently reported. However, the description of the electronic band structure of this QD system is still lacking.

In this paper, we investigate (In,Ga)As/GaP QDs in a low-indium-content range both from the theoretical and experimental points of view. The effects of both indium composition and QD geometry is analyzed through a combination of **k·p** and tight-binding (TB) simulations. Optical properties are then studied by temperature-dependent photoluminescence and time-resolved photoluminescence (TRPL).

## Methods

(In,Ga)As QDs are grown on n-doped GaP(001) substrate using gas-source molecular beam epitaxy. After the growth of a 450-nm buffer layer and a 4-monolayer (ML) (In,Ga)As deposition with 30 s of annealing under As (see the work of Nguyen Thanh et al. [13] for more information), a 30-nm GaP capping layer is finally deposited to prevent surface non-radiative recombinations. The growth temperature is set at 580°C. The nominal composition of indium is set at 30%, but because of high growth temperature, indium effective composition is assumed to be below or equal to 15%.

Temperature-dependent PL experiments are carried out by exciting samples with a 405-nm continuous-wave laser diode. Power density is roughly estimated at 80 W·cm^−2^. Samples are set in a helium bath closed-cycle cryostat to study PL from 10 K to room temperature. Measurements are also performed above room temperature using a hot plate. Attention is given to avoid the red luminescence of the deep centers in n-doped GaP substrates. Actually, the penetration length of the 405-nm beam is lower than the thickness of the GaP buffer layer, avoiding the substrate to be excited. Secondly, similar PL spectra are obtained on the same structures on non-doped GaP substrate, thus excluding any significant contribution from the GaP deep center luminescence.

For TRPL measurements, the sample is excited by a frequency-doubled Ti/sapphire laser at the wavelength of 405 nm. The repetition rate is 80 MHz. The PL signal is analyzed by an S20 streak camera, and measurements are performed at 10 K to overcome non-radiative recombinations channels.

## Results and discussion

### Band structure calculations

The eight-band **k·p** method has been extensively used to accurately simulate the electronic band structure of QDs with type I band alignment and direct optical transition (InAs/GaAs, InAs/InP…) [15,16]. The case of InGaAs/GaP QDs in the low-In-content range is expected to be trickier because of the coupling of zone center conduction band states with conduction band states located on the edge of the Brillouin zone [13]. To deal with this issue, we simulate the direct optical transition with the eight-band **k·p** method. To get an estimation of X-like and L-like state energies in the dot, we consider the TB *sp*^3^*d*^5^s* model [17] for a QW with a thickness equal to the height of the dot. Thus, the lateral quantum confinement effect is disregarded but is assumed to have negligible effects on lateral valleys with large effective masses.

**Figure 1:**
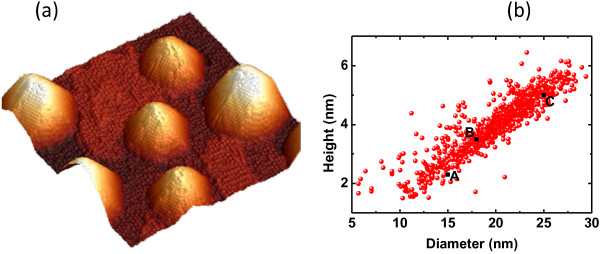
**(In,Ga)As QD image and statistical correlation.** (**a**) A 75 × 75-nm^2^ STM 3D plane view of (In,Ga)As QDs. (**b**) Statistical correlation between diameter and height on a 800 × 800-nm^2^ image.

**Figure 2:**
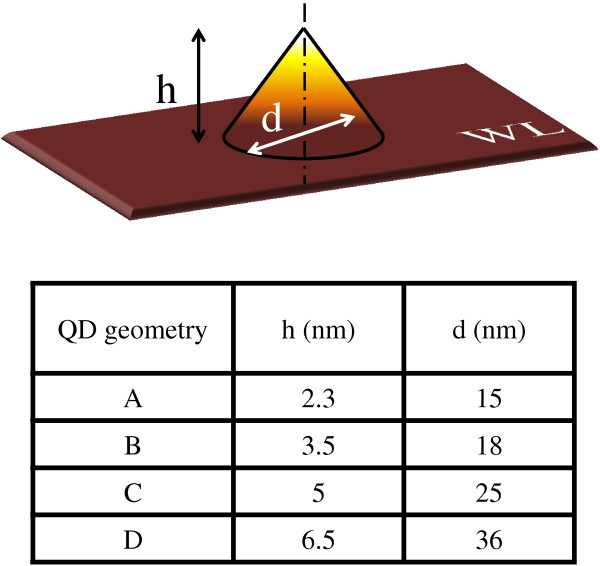
QD morphologies used for the eight-band k·p calculations.

To consider realistic QD geometries for the simulation, the morphology of InGaAs/GaP QDs are imaged by plane-view scanning tunneling microscopy (STM). The 75 × 75-nm^2^ STM image shown on Figure 1a exhibits InGaAs/GaP QDs with approximately a cone shape. The in-plane anisotropic ratio (between length and width) is indeed measured in the range of 1 to 1.5. The statistical analysis of diameter and height distributions is presented in Figure 1b. The **k·p** simulation is performed using the geometry defined on Figure 2. A *C*_∞v_ symmetry is considered for QD geometries, and strain calculations are performed using elasticity and parameters of Vurgaftman et al. [18] and the finite element method for numerical computation. Three typical dimension sets representative of the inhomogeneous size distributions are summarized in the table of Figure 2. The A, B, and C geometries correspond to real QDs typically found in the sample (see Figure 1b). The D geometry is chosen to study theoretically larger QDs in order to address the problem of lowering the emission energy. A typical wetting layer of 1-ML thick is added in the model to account for the Stranski-Krastanov growth mode. Deformation potentials and Luttinger parameters used in the **k·p** model are those extracted from the TB calculation for bulk InGaAs and GaP [17]. The valence band offsets are taken from recent *ab initio* calculations [19].

**Figure 3:**
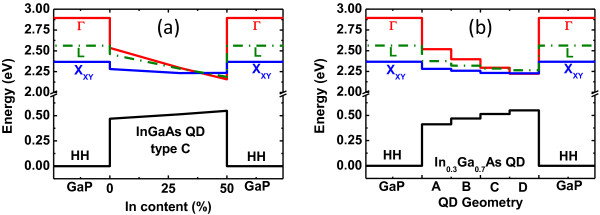
**Influence of In content and QD geometry on the electronic levels.** (**a**) Electronic levels of In_*x*_Ga_1−*x*_As QD with geometry C. (**b**) Electronic levels of In_0.3_Ga_0.7_As QDs for the four geometries defined in Figure 2. The Γ electronic level and the heavy-hole level in the QD are calculated with the k·p method. The X and L electronic levels are calculated with the TB model.

The influence of In content is presented on Figure 3a for a QD with the C geometry. The first electronic levels in the Γ, X, and L valleys and the first heavy-hole level of the QD are represented as a function of the In content. The electronic Γ and the heavy-hole levels are calculated with the **k·p** method, whereas the X and L electronic levels are calculated with the TB model. For low In content (below 30%), the ground optical transition is type I but is indirect with the first electron level of the X type. For very low In content (below 15%), the Γ-type conduction band level in the QD is even located at an energy above the one of the X-type conduction band of the GaP barrier. Nevertheless, a direct and type I ground state transition is predicted for In composition above 38%. This is a necessary condition in order to obtain a very efficient optical transition for such a QD geometry.

The influence of QD geometry is shown on Figure 3b for a medium In content of 30%. For small QDs, the first Γ electronic level undergoes an important quantum confinement effect which lifts up this level above both X and L levels, which are less affected by confinement effects. An indirect to direct type crossover is predicted for large QDs.

In conclusion, an increase of In content and an enlargement of QD size are expected to lower the first Γ-type conduction band level and thus yield an efficient optical transition. Moreover, the indirect to direct type crossover should be induced by strain relaxation associated to In content increase and QD enlargement.

### Optical properties

**Figure 4:**
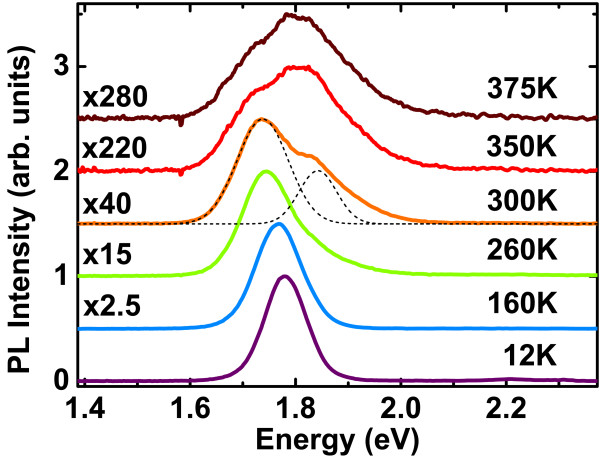
**Temperature-dependent PL spectra of (In,Ga)As/GaP QDs.** The black thin dashed lines show the fit of the two transitions by two Gaussian peaks.

Continuous-wave PL spectra are presented in Figure 4 for various temperatures. At 12 K, the PL spectrum exhibits a single peak centered at 1.78 eV. The peak shape undergoes a strong evolution from low to high temperatures. At 260 K, a shoulder appears on the high-energy side of the spectrum. At 300 K, a second optical transition clearly appears. When increasing the temperature above 300 K, the maximum of PL intensity switches from the low-energy (LE) transition to the higher-energy (HE) transition. This behavior may indicate that the HE optical transition is more efficient than the LE one. At 300 K, the LE transition is reported at 1.74 eV and the HE transition at 1.84 eV.

**Figure 5:**
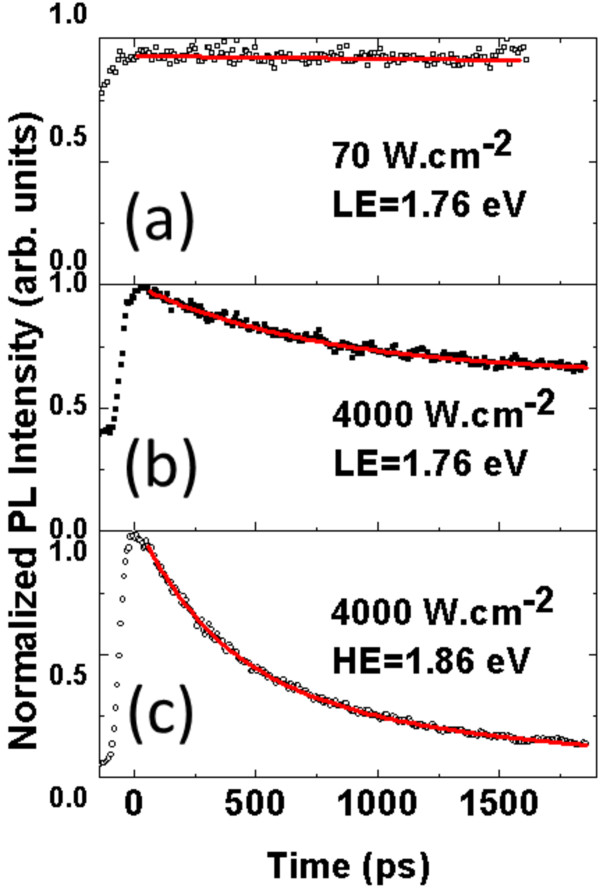
**PL dynamics at 10 K at selected energies, LE and HE.** For power densities of (**a**) 70 and (**b, c**) 4,000 W·cm^−2^. Red lines show biexponential fits.

To understand the nature of these two optical transitions, the dynamics of the recombination of carriers are investigated through TRPL spectroscopy. Experiments are performed at 10 K to overcome non-radiative recombination channels. The radiative lifetimes are deduced from the measured PL decay times. The sample is first excited with a low-incident power density equal to 70 W·cm^−2^. The LE optical transition is only detected in accordance with the spectrum shown on Figure 4 at low temperature. The evolution of the emission as a function of time is shown on Figure 5a. The LE optical transition exhibits a very long decay time which is greater than the repetition period of the laser (12 ns) and is not easily measurable with this experimental setup. Such a long lifetime can be interpreted on the basis of the theoretical results of the previous section. The energy position of the LE PL peak at low temperature (*E*_LE_ = 1.78 eV) is consistent with the calculated indirect transition (between 1.74 and 1.79 eV) for medium-sized dots in the 0% to 15% In content range.

The sample is then excited with a large power density equal to 4,000 W·cm^−2^ in order to fill the low-energy electronic levels and allow the HE optical transition to occur. The PL dynamics at selected energies, *E*_LE_ and *E*_HE_, are respectively shown on Figure 5b,c. The time-resolved emission related to the LE transition can be fitted by the sum of a shorter exponential decay with a lifetime of 770 ps and a constant associated with the very long lifetime of the indirect transition. Many-body Auger effects leading to an enhancement of intradot carrier relaxation may lower the optical transition lifetime. The density of electron-hole pairs is indeed estimated to be high (above 10 per QD). Such effects have been observed in InAs/InP QDs [20]. For the HE transition, the emission shows a biexponential decay with short lifetimes of 340 and 1,700 ps, respectively. Both times are consistent with a direct type-I electronic transition in QDs and a better overlap of electron and hole wave functions. The *E*_HE_*E*_LE_ difference is also in reasonable agreement with that of theoretical calculations. For large-sized dots and In content of 15%, an energy difference of 100 meV is indeed calculated between both direct and indirect optical transitions.

## Conclusions

The (In,Ga)As/GaP QD system is studied both theoretically and experimentally. The simulation results of **k·p** and TB methods are coupled and predict an indirect to direct crossover with the increase of In content and the ripening of QDs. Optical properties are then studied in the low-In-content range. In agreement with theoretical results, TRPL measurements are consistent with a ground optical transition of indirect type. A direct optical transition can be observed for high-power density or at room temperature where electrons get enough thermal energy to partially fill the Γ-type conduction band state.

## Competing interests

The authors declare that they have no competing interests.

## Authors' contributions

CR performed the optical property measurements and theoretical calculations. TNT, CC, and NB performed the MBE growth and the analyses of quantum dot geometry. PT and ST performed the STM measurements. MP, AB, HF, and XM supervised the measurements of optical properties. LP, MON, JE, and JMJ developed the simulation of electronic properties. OD and ALC managed the team. All authors read and approved the final manuscript.
